# Giant Renal Parapelvic Cyst: A Diagnostic Conundrum

**DOI:** 10.7759/cureus.99019

**Published:** 2025-12-12

**Authors:** Hemant Deshpande, Rohit S Deshpande

**Affiliations:** 1 Urology, Yashodhara Super Speciality Hospital Unit 2, Solapur, IND; 2 Urology, Medanta - The Medicity, Gurugram, Gurugram, IND

**Keywords:** enterogenous cyst, giant hydronephrosis, mesenteric cyst, parapelvic cyst, renal cyst

## Abstract

Renal cysts are the most common lesions of the kidney and affect a significant proportion of all individuals undergoing renal imaging. They can be unilateral/bilateral, focal/multifocal, or acquired/congenital. Parapelvic cysts, a subset of renal cysts, are uncommonly encountered in clinical practice. Parapelvic cysts progressing in size to involve almost the entire abdomen are even rarer. Here, we report a case of a giant parapelvic cyst in a middle-aged Asian gentleman, which was managed successfully with open drainage and excision, and is one of the largest parapelvic cysts ever reported in the medical literature.

## Introduction

Renal cysts affect 20-50% of the population. Most renal cysts are asymptomatic, incidentally detected, and rarely require surgical treatment [1]. They may be simple, complex, or part of systemic diseases such as polycystic kidney disease. Simple cysts are usually incidental, while complex cysts may present with pain, hematuria, infection, or hypertension. Ultrasound can help identify simple versus complex cysts, depending on cyst contents, echogenicity, and vascularity. Contrast-enhanced CT is the gold standard for Bosniak classification, and MRI offers better soft-tissue delineation and assessment. Intervention is indicated for symptomatic cysts, infected/hemorrhagic cysts, obstruction, or Bosniak III-IV complex cysts where risk of malignancy warrants surgical management.

Parapelvic cysts represent 1-2% of renal cysts and are more likely to compress critical structures (near the renal hilum) and thereby are more likely to cause symptoms due primarily to their location. They do not communicate with the renal collecting system and are attributed variably by some to be lymphatic in origin or as embryological remnants of the developing mesonephros [2]. They can be asymptomatic or can present with symptoms ranging from a dull-aching pain, hematuria, to backpressure symptoms (due to compression of the collecting system) on the affected kidney (sometimes causing renal functional deterioration) [3]. Here, we present a case of a middle-aged gentleman with a huge symptomatic parapelvic cyst, necessitating prompt treatment.

## Case presentation

**Figure 1:**
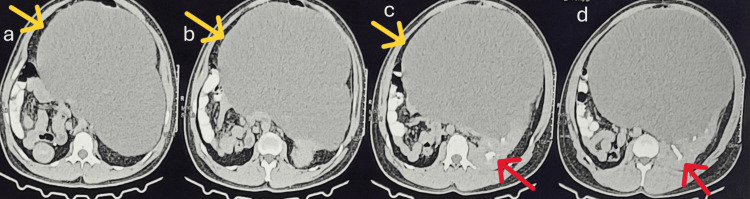
Axial contrast-enhanced CT images. (a-c) Extension (marked by yellow arrows) of the parapelvic cyst to the contralateral side of the abdomen. (c) Contrast opacification (marked by a red arrow) of the pelvicalyceal system. (d) The proximity of the cyst wall to the pelvicalyceal system (marked by a red arrow) shows no contrast within the cyst cavity, thereby confirming the non-communicating nature of the cyst.

**Figure 2:**
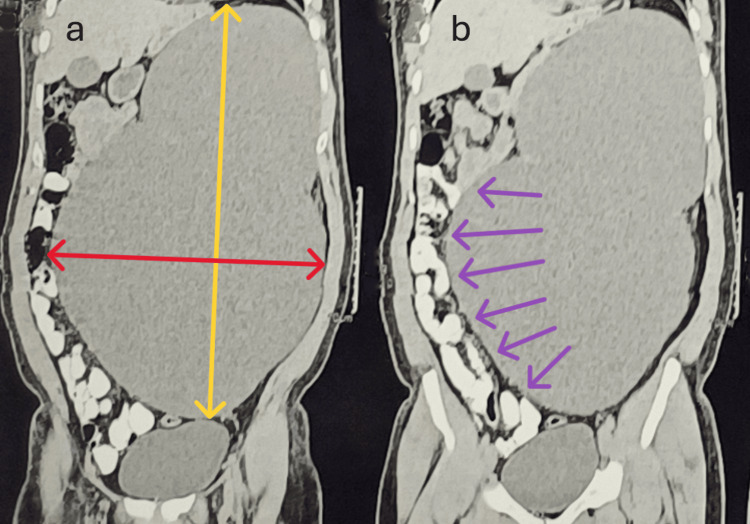
Coronal sections of CT. (a) Vertical (marked by a bidirectional yellow line) and horizontal (marked by a bidirectional red line) extent of the parapelvic cyst. (b) The displacement of the bowel loops to the contralateral hemiabdomen (marked by purple arrows) caused by the cyst wall.

**Figure 3:**
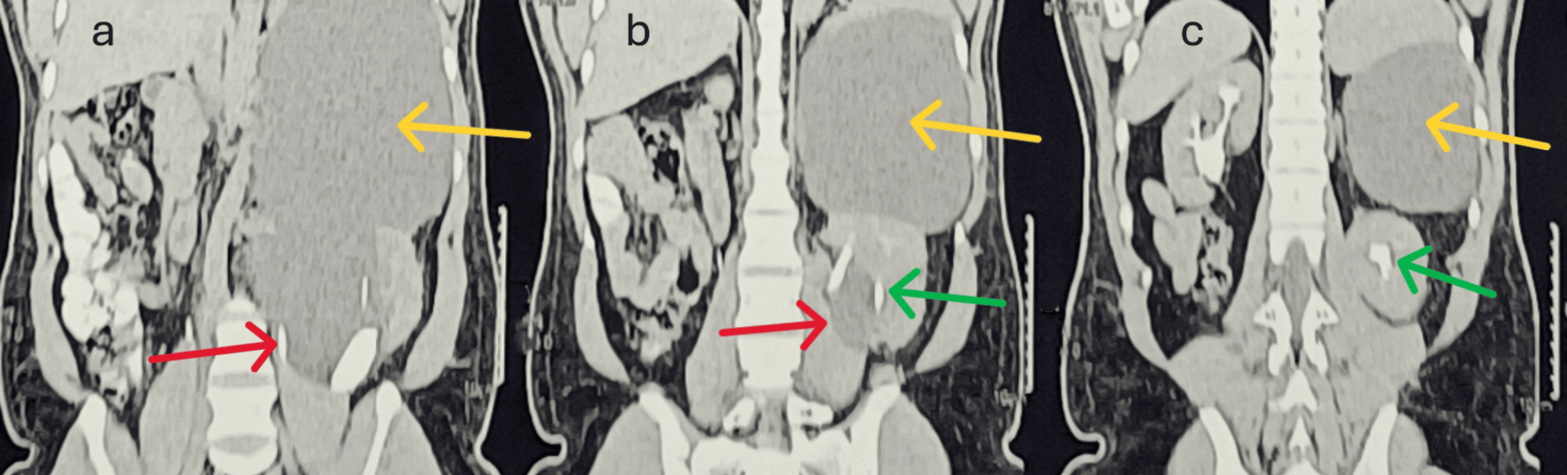
Coronal sections of CT demonstrating the origin and extensions of the parapelvic cyst. (a-c) The origin of the parapelvic cyst from the hilum (marked by red arrows in a and b) of the kidney and the superior extensions (marked by yellow arrows) of the parapelvic cyst pushing the kidney downwards. (b, c) Contrast opacification in the excretory phase, and infundibular splaying of the collecting system (marked by green arrows) due to the parapelvic cyst.

A 38-year-old Asian gentleman presented to the outpatient department with progressive abdominal distension and dull-aching left flank pain. Ultrasound was performed initially, and he was diagnosed with left-sided gross hydronephrosis with papery-thin left renal cortex. Subsequent contrast-enhanced CT of the abdomen and pelvis (with delayed films) revealed a left-sided large renal parapelvic cyst measuring 38 × 27 × 20 cm (craniocaudal × transverse × anteroposterior), occupying an area more than the hemi-abdomen and extending down to the pelvis (Figures 1-3) with well-preserved left renal cortical thickness and prompt contrast excretion in both the kidneys.

**Figure 4:**
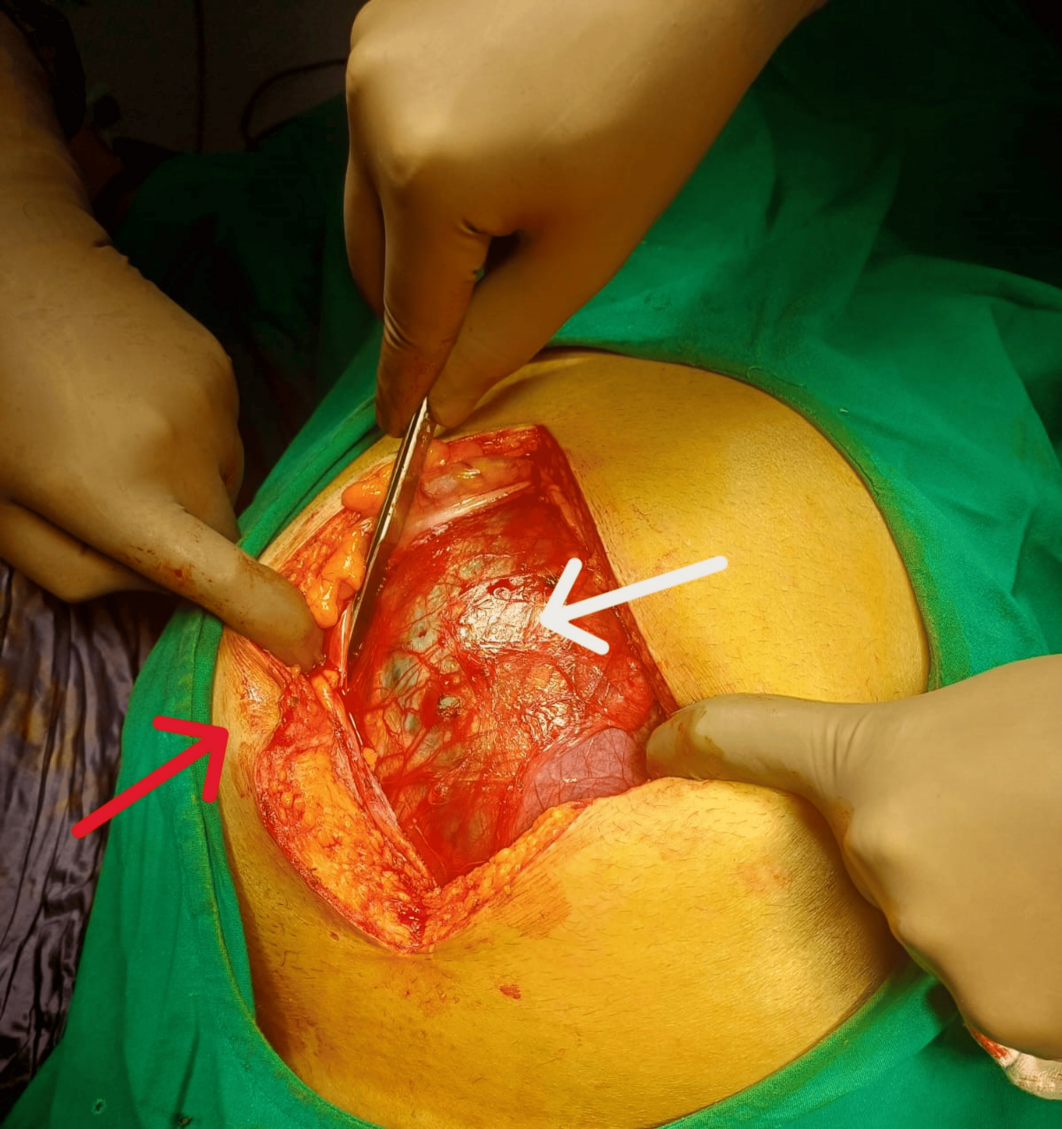
Initial appearance of the cyst. Initial appearance of the cyst (cyst wall shown by a white arrow) after opening the abdomen (via a midline periumbilical incision) (umbilicus shown by a red arrow).

In view of the large size of the parapelvic cyst and keeping in mind the differential diagnosis of a large mesenteric or enterogenous cyst, an open approach was chosen via a midline incision (centered around the umbilicus) (Figure 4).

**Figure 5:**
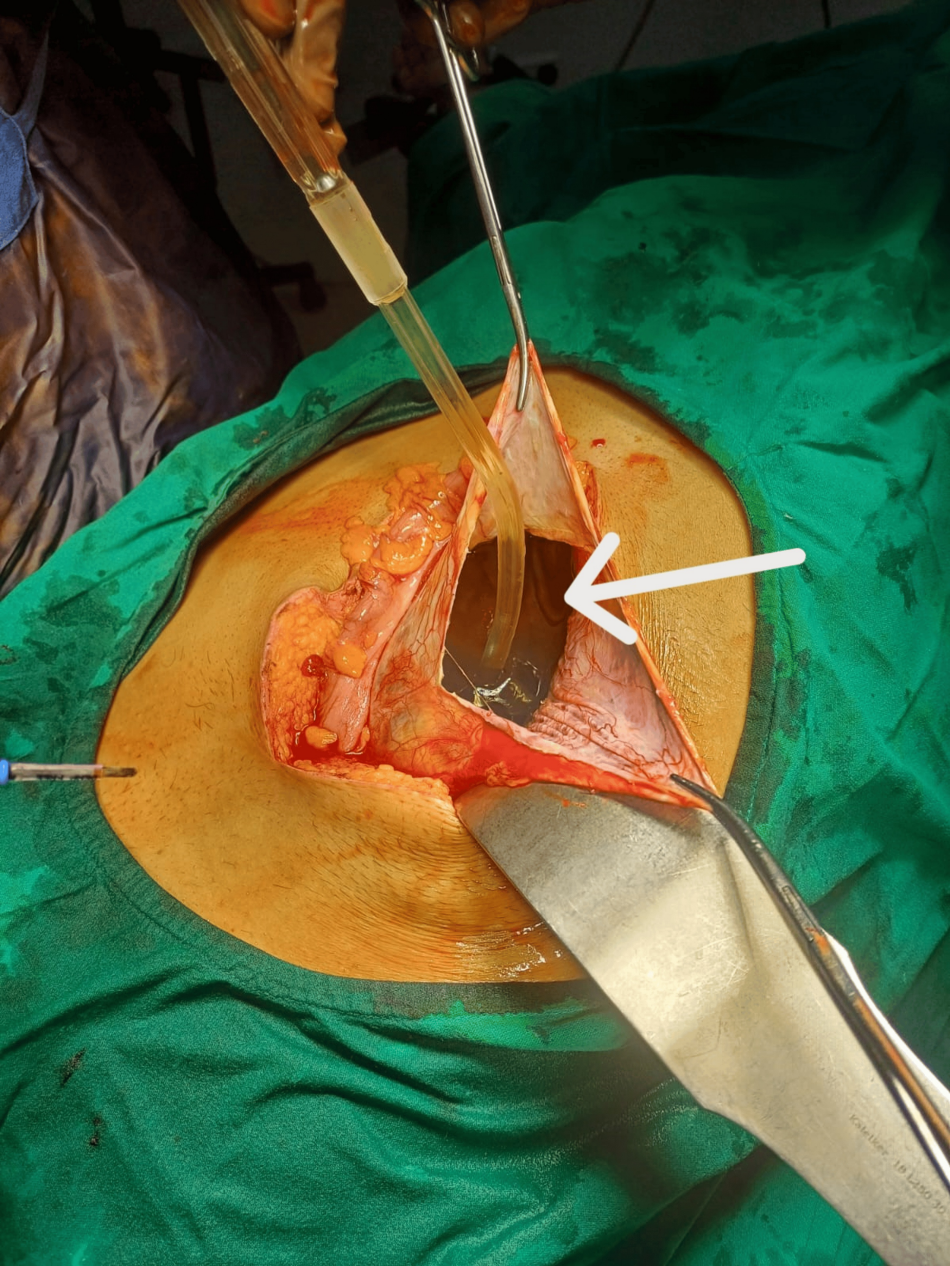
Clear fluid (shown by a white arrow) (approximately 6 L) aspirated from the cyst cavity.

After reflecting the descending colon, the cyst was opened, approximately 6 L of clear fluid was drained (Figure 5), and the cyst wall was excised completely.

**Figure 6:**
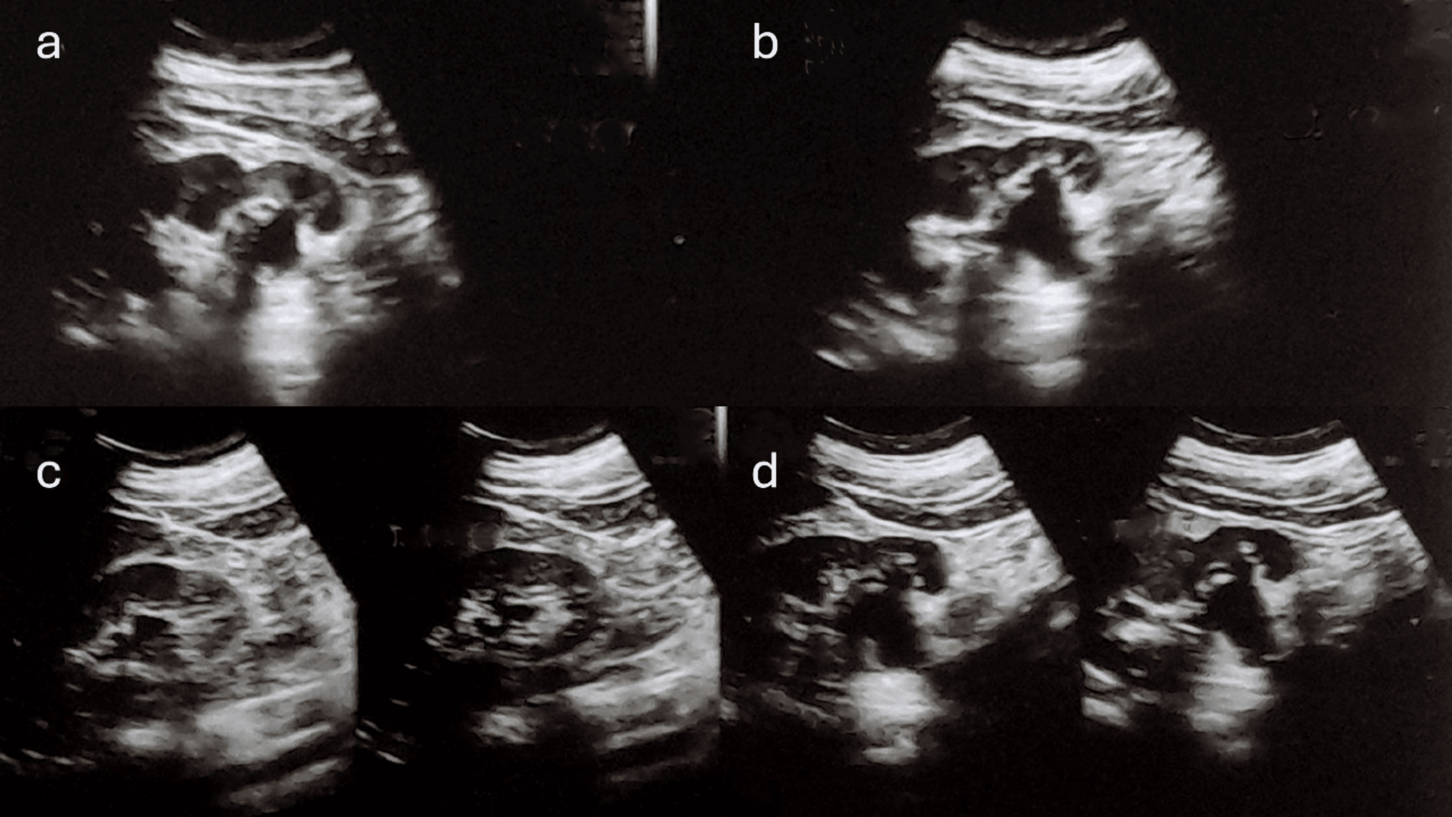
Postoperative ultrasound demonstrating normal-looking bilateral kidneys (a, b, d: left kidney; c: right kidney).

Patient recovered well, was relieved of his symptoms, and was discharged on the second postoperative day. Postoperative ultrasound revealed normal bilateral kidneys (Figure 6).

## Discussion

Large cystic masses in the abdomen can have a myriad of presentations (ranging from abdominal pain to hematuria), and it can be challenging to localize the origin of such lesions. Imaging may help identify the organ of origin in these cases. While definitive diagnosis is not always possible with imaging, careful assessment of the imaging appearance, location, and relationship to adjacent structures can help narrow the differential diagnosis [4].

In the case of large cystic lesions of the kidney, often displacing the bowel to the opposite side, the differential diagnosis can also be that of a large mesenteric or enterogenous cyst, pelviureteric junction obstruction, or a large hydronephrotic kidney. Mao et al. [5] highlighted the importance of axial contrast imaging in differentiating parapelvic cysts from hydronephrosis. If the diagnosis is in doubt even after imaging, surgical exploration should be undertaken in the face of persistent symptoms.

Surgical approaches for parapelvic cysts include open or laparoscopic deroofing/excision and endoscopic laser incision. Laparoscopic deroofing/excision can be challenging, especially in large cysts (wherein an open approach may be preferred), and mandates the requirement of technical expertise. Endoscopic laser incision can be accomplished via ureteroscopy [6] (especially for the concomitant treatment of stones), using holmium laser; however, it is difficult to achieve hemostasis, remaining ineffective in treating cysts with thick walls [7]. Recently, greenlight laser has been found to be effective in achieving hemostasis in such cases in a retrospective analysis comparing greenlight and holmium lasers for ureteroscopy and endotomy of parapelvic cysts [8]. However, laser endotomy still carries the risk of catastrophic intraoperative hemorrhage due to inadvertent entry into neighboring blood vessels.

Other treatments described for parapelvic cysts include laparoscopic cyst decortication, ultrasound-guided puncture and instillation of sclerosants, single-time aspiration, and observation. However, ultrasound-guided puncture and aspiration are not without drawbacks and carry a risk of recurrence (22.8-30%) [2] or inadvertent entry into major vessels. Other complications include retroperitoneal leak of sclerosant and peritoneal inflammation.

## Conclusions

Parapelvic cysts are challenging to diagnose on ultrasound; however, axial imaging with contrast may prove to be an invaluable adjunct, especially due to splaying of the collecting system due to the mass effect of the cyst. Symptomatic parapelvic cysts warrant treatment, and the treatment approach may be tailored to the particular circumstances, depending on surgical expertise and technical proficiency.
